# The Deterioration of Vision during School Life

**Published:** 1904-07-16

**Authors:** 


### THE DETERIORATION OF VISION DURING SCHOOL LIFE.

In a paper read at the International Congress of School Hygiene at Nuremberg, Dr. Ettie Sayer[1] reported that the result of an investigation into the condition of the eyesight of children attending the elementary schools in London showed that 10 per cent. of the boys and 11 per cent. of the girls had a visual acuity of $\frac{6}{18}$ or less. These figures refer to children from 8 to 14 years of age. When the tests were applied to younger children, that is, from 6 to 8 years of age, it was found that 81 per cent. could read $\frac{6}{6}$ with each eye separately, and only in 3·5 per cent. were there defects sufficiently serious to reduce the vision as low as $\frac{6}{18}$. That is, stated broadly, the serious defects among children of 6 years of age are only 3 per cent., whereas at 11 years the proportion has reached 11 per cent., after which the figures are somewhat less. To avoid this deterioration of vision during school life, Dr. Sayer suggests that all children should be tested at the outset, and that where vision is below the normal standard atropine should be employed and the refraction estimated. When there is excessive hypermetropia *plus* glasses should be worn until this is outgrown. Commencing myopia raises the question whether the child should be allowed to enter on any form of near work, and in any event a special curriculum with a minimum of near work should be ordered. All young children should be preserved from small type and small writing. As the law demands compulsory education, it ought to provide means of protection for the many thousands of children whose eyesight and general health are prejudiced by the effort of accommodation involved in learning lessons for many hours every day of their school lives.

Another contribution bearing upon the effect of prolonged near work on vision comes from the anthropometrical laboratory of the University of Aberdeen.[2] Dr. G. Stoddart and Dr. C. H. Usher have conducted a series of observations on the eyes of 400 students and have tabulated the results. From these it appears that as regards acuteness of vision the Aberdeen students compare favourably with the students of certain foreign universities, and that in particular the proportion of myopes is much smaller. Thus in Aberdeen these numbered 18·7 per cent., as compared with 28 per cent. in some American universities, and 59 per cent. at the University of Breslau. An analysis of the observations also shows that myopes are more frequent in town-bred than in country-bred, students. Contrary to the general creed the average size of the pupil in the hypermetropes was found to be larger than in the myopes. Light-coloured eyes were found to have a slightly larger pupil than dark or grey-coloured eyes. Acuteness of vision did not appear to have any definite relationship either to size of pupil or colour of iris. Nearly 3 per cent. of the students tested were found to be colour-blind.

[1] Brit. Med. Journ., June 18th, 1904. [2] Lancet, June 4th, 1904.

